# An Unusual Association of Idiopathic Syndrome of Inappropriate Antidiuretic Hormone Secretion (SIADH) With Interstitial Lung Disease (ILD)

**DOI:** 10.7759/cureus.48304

**Published:** 2023-11-05

**Authors:** Shubhashis Saha, Tias Saha, Srishti Sharma, Nader Mahmood

**Affiliations:** 1 Internal Medicine, Christian Medical College Vellore, Vellore, IND; 2 Internal Medicine, Diabetic Association Medical College, Faridpur, BGD; 3 Internal Medicine, Saraswati Medical College, Unnao, IND; 4 Pulmonary Medicine, St. Mary’s General Hospital, Passaic, USA

**Keywords:** hyponatremia, syndrome of inappropriate adh secretion, siadh, interstitial lung disease, ild

## Abstract

Although the syndrome of inappropriate antidiuretic hormone secretion (SIADH) is commonly associated with many lung conditions and drugs used for treating them, no literature describes a direct association between SIADH and interstitial lung disease. This case report discusses a 79-year-old male patient who presented to the emergency department (ED) with altered mental status following a fall. The patient had clinical symptoms and imaging findings concerning interstitial lung disease (ILD), and laboratory tests from the ER indicated severe hyponatremia and an increased white blood cell count, suggesting an unusual clinical picture. Detailed workup and medication reconciliation revealed no other medical conditions or intake of drugs associated with SIADH; however, the patient’s low serum osmolality, high urine osmolality, high urine sodium, and improvement in serum sodium level with the initiation of 0.9% saline, salt tablets, and tolvaptan verify the presence of SIADH. While the association between SIADH and ILD is not well documented in medical literature, a few case reports from different regions have indicated a potential link, either through drug-induced ILD or SIADH resolution coinciding with ILD improvement. Hence, we describe a case of idiopathic SIADH, possibly associated with interstitial lung disease. This case demonstrates the importance of recognizing the coexistence of SIADH and ILD, as severe hyponatremia can lead to potential life-threatening neurological consequences.

## Introduction

Syndrome of inappropriate antidiuretic hormone secretion (SIADH) is a distinct clinical entity characterized by the excessive and unregulated secretion of antidiuretic hormone (ADH). This hormone plays a crucial role in regulating water balance within the body by promoting water reabsorption in the kidneys. In SIADH, excessive ADH leads to increased water retention and concentrated urine, resulting in hyponatremia [1].

Interstitial lung disease (ILD) encompasses a heterogeneous group of disorders characterized by inflammation and fibrosis of the lung parenchyma. This condition results in impaired gas exchange, leading to reduced lung function and, in severe cases, respiratory failure.

While the exact prevalence and underlying association of SIADH in patients with ILD is not well defined, its occurrence is infrequent and often overlooked due to its subtle and nonspecific symptoms. However, the coexistence of these two conditions can lead to complex and challenging clinical presentations, as both ILD and SIADH independently impact pulmonary and systemic physiology.

## Case presentation

History and examination

Our patient, a 79-year-old male, was brought to the emergency department (ED) by neighbors with altered mental status after he tripped and fell outside his house. He lost consciousness for about 15 minutes but denied any complaints of palpitations, nausea, or vomiting prior to the episode.

He had complaints of shortness of breath, cough, and wheezing for the last six months, for which he was evaluated at a primary care clinic two weeks prior. The initial chest X-ray showed bilateral reticular lung infiltrates, which was suspicious for interstitial lung disease; hence, a chest high-resolution computed tomography was scheduled in a month’s time. His other comorbidities were essential hypertension and benign prostatic hyperplasia, for which he was prescribed hydrochlorothiazide, lisinopril, and tamsulosin.

The patient was presently unemployed, lived alone, and had a poor social support structure. There was no significant occupational exposure or history of smoking or drug abuse. He did not have any family members with chronic lung conditions.

In the ED, his vitals were normal, with a temperature of 98.9°F, heart rate of 96 beats/min, blood pressure of 127/64 mmHg, respiratory rate of 22 breaths/min and oxygen saturation of 94% on room air. On examination, he has altered mental status and dry mucus membranes. The rest of the neurological examination was normal. He had bruises over his left shoulder and a laceration on the left side of his head, both sustained from the fall. Pulmonary examination showed the presence of respiratory distress with no stridor and wheezing but rales present. Examination of the other organ systems was normal.

Laboratory investigations and imaging studies

Routine laboratory investigations performed at the emergency department are shown below (Table 1). In addition, an arterial blood gas performed on supplemental oxygen of 2 L/min revealed a compensated respiratory acidosis with metabolic compensation.

**Table 1:** Initial laboratory results from the emergency department on day 1 pCO_2_: partial pressure of carbon dioxide in arterial blood, HCO_3_^-^: bicarbonate levels in arterial blood, pO_2_: partial pressure of oxygen in arterial blood, SO_2_: saturation of oxygen in arterial blood.

Parameters	Patient's result	Reference range
Serum sodium	111 mmol/L	135 - 145 mmol/L
Urine sodium	24 mmol/L	28 - 272 mmol/L
Serum osmolality	250 mOsm/Kg	285 - 295 mOsm/Kg
Urine osmolality	353 mOsm/Kg	50 -1400 mOsm/Kg
Serum chloride	75 mmol/L	95 - 105 mmol/L
Hemoglobin	13.4 g/dl	13.5 - 17.5 g/dl
WBC count	21000 cells/µL	4000 - 11000 cells/µL
Neutrophil count	86%	40 - 80%
Lactic acid (venous)	2.6 mg/dl	0.5 - 2.2 mg/dl
pH	7.39	7.35 - 7.45
pCO_2_	67.7 mmHg	35 - 40 mmHg
HCO_3_^-^	35.7 mmol/L	22 - 26 mmol/L
pO_2_	166.6 mmHg	80 - 100 mmHg
SO_2_	98%	94 - 100%

**Figure 1:**
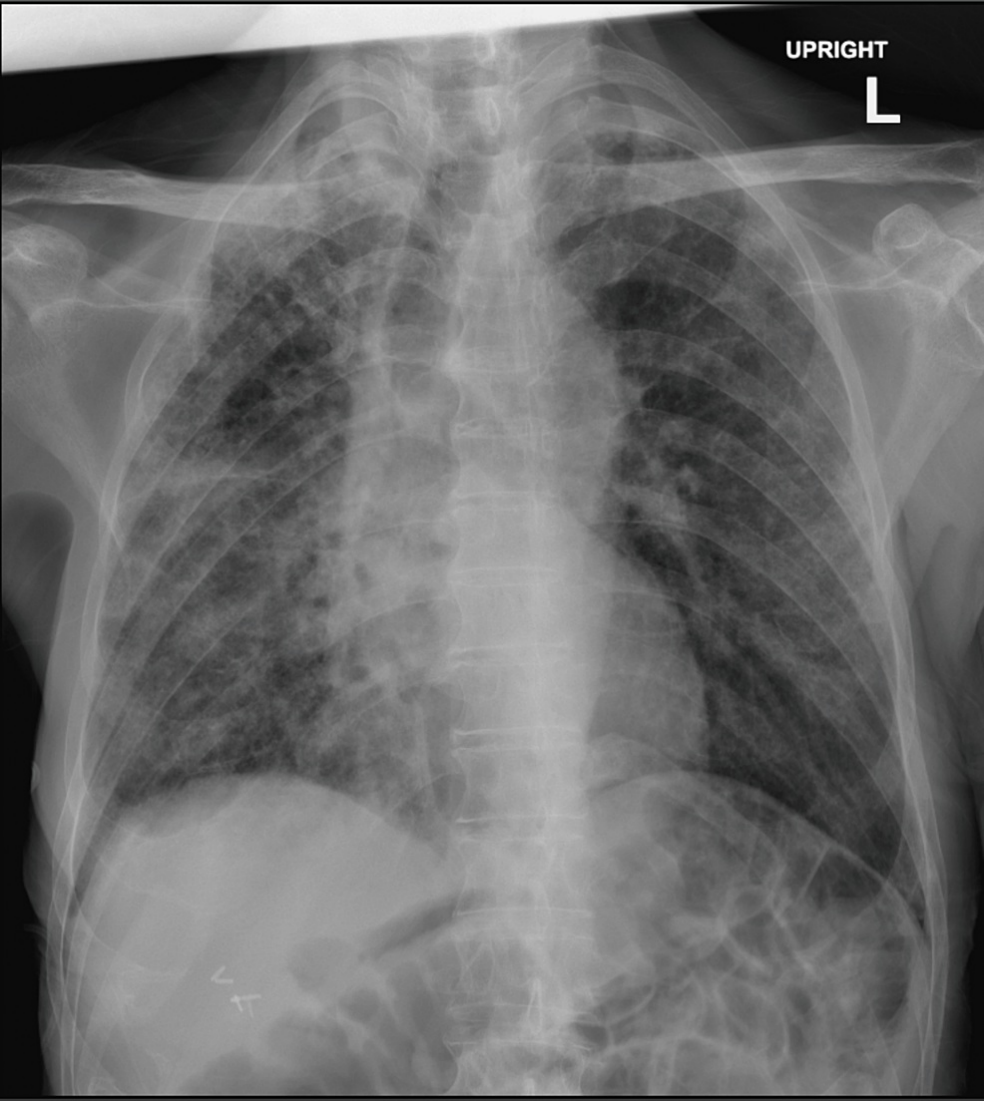
Chest X-ray performed at admission The image shows reticular opacity in the bilateral lung field.

**Figure 2:**
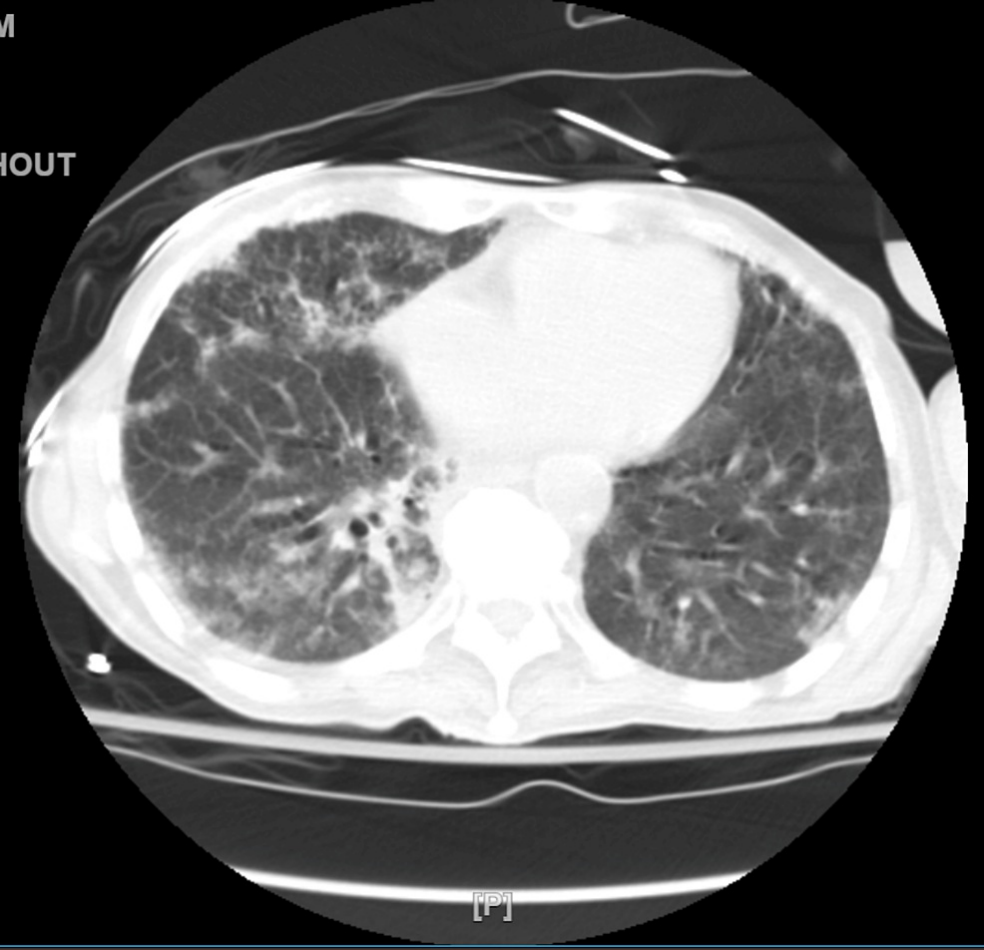
High-resolution CECT chest performed on day 1 of the hospital stay The image shows diffuse bilateral reticular ground glass opacities in the lower lung lobes. CECT: contrast-enhanced computed tomography

Chest X-ray revealed reticular opacity with bilateral lung infiltrate (Figure 1). A chest CT scan on day 1 of admission showed diffuse reticular ground-glass opacity (Figure 2). X-ray of the left shoulder showed a left humeral neck fracture.

Treatment

**Figure 3:**
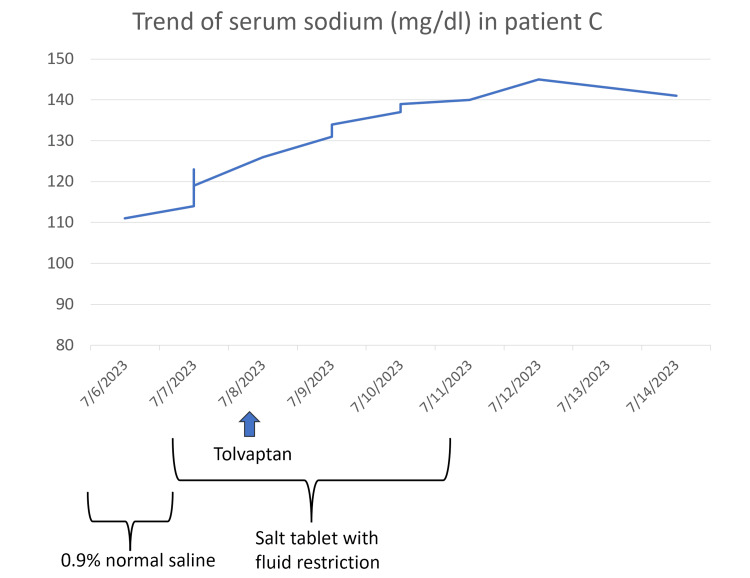
The trend of serum sodium (in mg/dl) in the patient during his hospital stay

Acetaminophen for pain, 0.9% saline for fluid resuscitation, and empiric antibiotics of ceftriaxone and azithromycin were administered in the ED. He was continued on normal saline maintenance fluids until the morning of day 2, with a minimal interval increase in serum sodium levels. Hence, a provisional diagnosis of possible SIADH was made, and fluid restriction with oral salt tablets was administered from the evening of day 2. The patient's sodium levels subsequently showed an increasing trend with adequate per-day sodium correction of 8 mg/dl/day; hence, oral sodium replacements at 1 g tablets thrice daily and fluid restriction were continued. A single dose of tolvaptan 15 mg was administered on day 2, but it was withheld subsequently due to a rapid increase in serum sodium levels. Additionally, the patient had CT chest evidence of traction-bronchiectasis on both lung apices, possibly from the usual interstitial pneumonia (UIP) variant of ILD, and an elevated WBC count, for which he was started on azithromycin 500 mg intravenous (IV) on day 1, and his WBC normalized to 9000 mg/dl by day 8. The patient’s symptoms improved gradually, his serum sodium normalized over a week (Figure 3), and he was discharged with acetaminophen and tamsulosin, with frequent outpatient follow-up visits scheduled to monitor his serum sodium levels.

## Discussion

The syndrome of inappropriate ADH release has been associated with many etiologies. Common etiologies are malignancies (small cell lung cancer, extrapulmonary small cell lung cancer, head and neck cancer), central nervous system (CNS) disturbances (including intracranial hemorrhage, stroke, and infections), and a long list of possibly implicated drugs [2].

In our patient with severe hyponatremia, initial laboratory results showed a patient with low serum osmolality and impaired renal water excretion, demonstrated by the increased urinary osmolality and elevated urinary sodium levels, thus being strongly suggestive of the diagnosis of SIADH. Treatment of hyponatremia was initiated with isotonic saline based on initial clinical appearance but later changed to oral salt tablets with fluid restriction in view of a minimal rise in serum sodium levels, which eventually normalized by the end of one week after admission.

The etiological workup for SIADH included a chest CT, which showed bilateral ground-glass opacity, which, with his symptoms of progressive cough and dyspnea, was suggestive of interstitial lung disease. CT brain was performed due to his presentation with head trauma, which was normal with no signs of intracranial hemorrhage. Serum and urine analyses were negative for signs of infection. Hypothyroidism and hypocortisolism were ruled out as potential causes of hyponatremia as well. An extensive medication review revealed no current medications that could cause SIADH as a rare adverse event. Hence, a diagnosis of idiopathic SIADH was made, which has been shown to be more common in the elderly population, similar to our patient. Although idiopathic SIADH has been postulated to be related to the aging process [3,4], multiple studies have considered it a catch-all basket for SIADH of unknown etiology whose underlying cause may ultimately be revealed on a subsequent visit, for example, from an occult malignancy.

SIADH has been implicated in many lung pathologies, including lung malignancies, pneumonia, and antifibrotic medications like pirfenidone [5] for the management of ILD. However, only a few case reports have described a direct association between ILD and SIADH. One case report from Japan described a patient with interstitial pneumonia superimposed on pneumoconiosis who developed SIADH, whose resolution temporally correlated with radiological and clinical improvement of the ILD after starting high-dose prednisolone [6]. Another case report from Japan showed a similar temporal correlation between the resolution of acute interstitial pneumonia and that of SIADH [7]. Two cases, one each from Korea [8] and Japan [9], have described ILD caused by known SIADH-associated drugs, carbamazepine and cyclophosphamide, respectively, which ultimately caused SIADH. In our patient with essentially an idiopathic SIADH coinciding with a newly diagnosed interstitial lung disease and in the absence of any known causes of SIADH, an association between the two can be argued in line with the myriad mechanisms proposed to explain lung pathologies triggering SIADH [10].

## Conclusions

This case report presents a possible association between ILD and SIADH. Since severe hyponatremia can lead to life-threatening neurological complications, understanding the possible association between SIADH and ILD is essential for recognizing the potential coexistence of these two conditions in clinical practice. Further research into the epidemiology and mechanisms is required to establish a clear relationship between ILD and SIADH. Additionally, raising awareness among clinicians about the potential association could aid in the early detection and management of SIADH in patients with ILD, ultimately improving patient outcomes.
